# All-passive transformable optical mapping near-eye display

**DOI:** 10.1038/s41598-019-42507-0

**Published:** 2019-04-15

**Authors:** Wei Cui, Liang Gao

**Affiliations:** 1 Department of Electrical and Computer Engineering, University of Illinois at Urbana-Champaign, 306 N. Wright St., Urbana, 61801 IL USA; 2 Beckman Institute for Advanced Science and Technology, University of Illinois at Urbana-Champaign, 405 N. Mathews Ave., Urbana, 61801 IL USA

## Abstract

We present an all-passive, transformable optical mapping (ATOM) near-eye display based on the “human-centric design” principle. By employing a diffractive optical element, a distorted grating, the ATOM display can project different portions of a two-dimensional display screen to various depths, rendering a real three-dimensional image with correct focus cues. Thanks to its all-passive optical mapping architecture, the ATOM display features a reduced form factor and low power consumption. Moreover, the system can readily switch between a real-three-dimensional and a high-resolution two-dimensional display mode, providing task-tailored viewing experience for a variety of VR/AR applications.

## Introduction

The emergence of virtual reality (VR)/augmented reality (AR) technologies opens a new way that people access digital information. Despite tremendous advancement, currently, very few VR/AR devices are crafted to comply with the “human-centric design” principle^1^, a rising consensus that the hardware design should center around the human perception^2^. To meet this standard, a near-eye display must integrate displays, sensors, and processors in a compact enclosure, while allowing for a user-friendly human-computer interaction. Among these four pillar requirements, the display plays a central role in creating a three-dimensional perception^3^ that mimics real-world objects.

Conventional near-eye three-dimensional displays are primarily based on computer stereoscopy^4^, creating depth perception using images with parallax for binocular vision. These two-dimensional images with parallax are combined to yield three-dimensional representations of the scenes, namely binocular disparity cues. A long-standing problem in computer stereoscopy is the vergence-accommodation conflict^5^. In this scenario, the viewer is forced to adapt to conflicting cues between the display and the real world, causing discomfort and fatigue. This problem originates from the mismatch between the fixed depth of the display screen (*i.e*., accommodation distance) and the depths of the depicted scenes (*i.e*., vergence distance) specified by the focus cues. This mismatch contradicts the viewer’s real world experience where these two distances are always identical.

To mitigate the vergence-accommodation conflict, there are generally two strategies^6^. The first strategy, referred to as temporal multiplexing (varifocal)^7–12^, rapidly sweeps the focal plane of the projector either mechanically or electronically. Mechanical sweeping is normally performed either through adjusting the optical power of the eyepiece (e.g., an acoustic lens^13–15^, a birefringent lens^16^ or an Alvarez lens^17^) or simply shifting the axial distance between the display and the eyepiece^18^. While the electronic sweeping of the focal plane can be accomplished by using various active optical devices, such as a liquid crystal lens and a deformable mirror device^19–21^. Moreover, the varifocal focal effect can also be created by using multiple layers of spatial light modulators with directional backlighting^22^ and high-speed shutters^23^. In temporal-multiplexing-based displays, a series of two-dimensional images are presented sequentially at varied depths by using high-speed display devices, such as a digital micromirror device (DMD). Despite a high resolution, the temporal-multiplexing-based methods are limited by the fact that product of the image dynamic range, depth plane number and the volumetric display rate cannot be greater than the maximum binary pattern rate of the DMD. For example, given a typical DMD’s maximum binary pattern rate at 20 kHz and six depth planes displayed at a volumetric image refresh rate at 60 Hz, the dynamic range of each image is limited to only 6 bits (64 grey levels). The second strategy, referred to as spatial multiplexing^24–26^, optically combines multiple panel images and simultaneously project them towards either different depth planes (multifocal) or perspective angles (light field) using devices such as a beam splitter^27^, a freeform prism^28^, a lenslet array^29–31^, a pinhole array^32^, a holographic optical element (HOE)^33,34^ or a liquid-crystal-on-silicon spatial light modulator (LCOS-SLM)^35,36^. Compared with temporal multiplexing, the spatial-multiplexing-based methods have an edge in image dynamic range. Nonetheless, the current implementations are plagued by various problems. For example, using beam splitters usually leads to a bulky setup, making it unsuitable for wearable applications. The lenslet-array-based display (*i.e*., the light field display) suffers from a low lateral resolution (102 × 102 pixels^37^; 146 × 78 pixels^38^) due to the trade-off between the spatial and angular resolution^39–41^. In holographic displays, although the screen resolution and the number of perspective angles are decoupled, the image quality is generally jeopardized by the speckle noise^42^. Moreover, the calculation of holographic patterns is computationally prohibitive, restricting their use in real-time display applications^43–45^. Lastly, the LCOS-SLM-based approach relies on an active optical component (LCOS-SLM) to execute its core function, unfavorably increasing power consumption and the device’s form factor^46^.

To enable a compact near-eye three-dimensional display featuring both high resolution and image dynamic range, herein we developed an all-passive, transformable optical mapping (ATOM) method. Like the LCOS-SLM-based approach, the ATOM display is based on spatial multiplexing—it simultaneously maps different parts of a two-dimensional display screen to varied depths. Therefore, the product of the lateral resolution and the number of depth planes equates the total number of display pixels at the input end. However, rather than using the LCOS-SLM, the ATOM display employs a passive diffractive optical element—a distorted grating—to achieve two-dimensional-to-three-dimensional mapping. This innovative all-passive optical architecture significantly reduces the power consumption and the device’s form factor. Moreover, to improve the device’s usability, we build the system on a transformable architecture which allows a simple switch between the three-dimensional and high-resolution two-dimensional display modes, providing task-tailored viewing experience.

## Operating Principle

**Figure 1:**
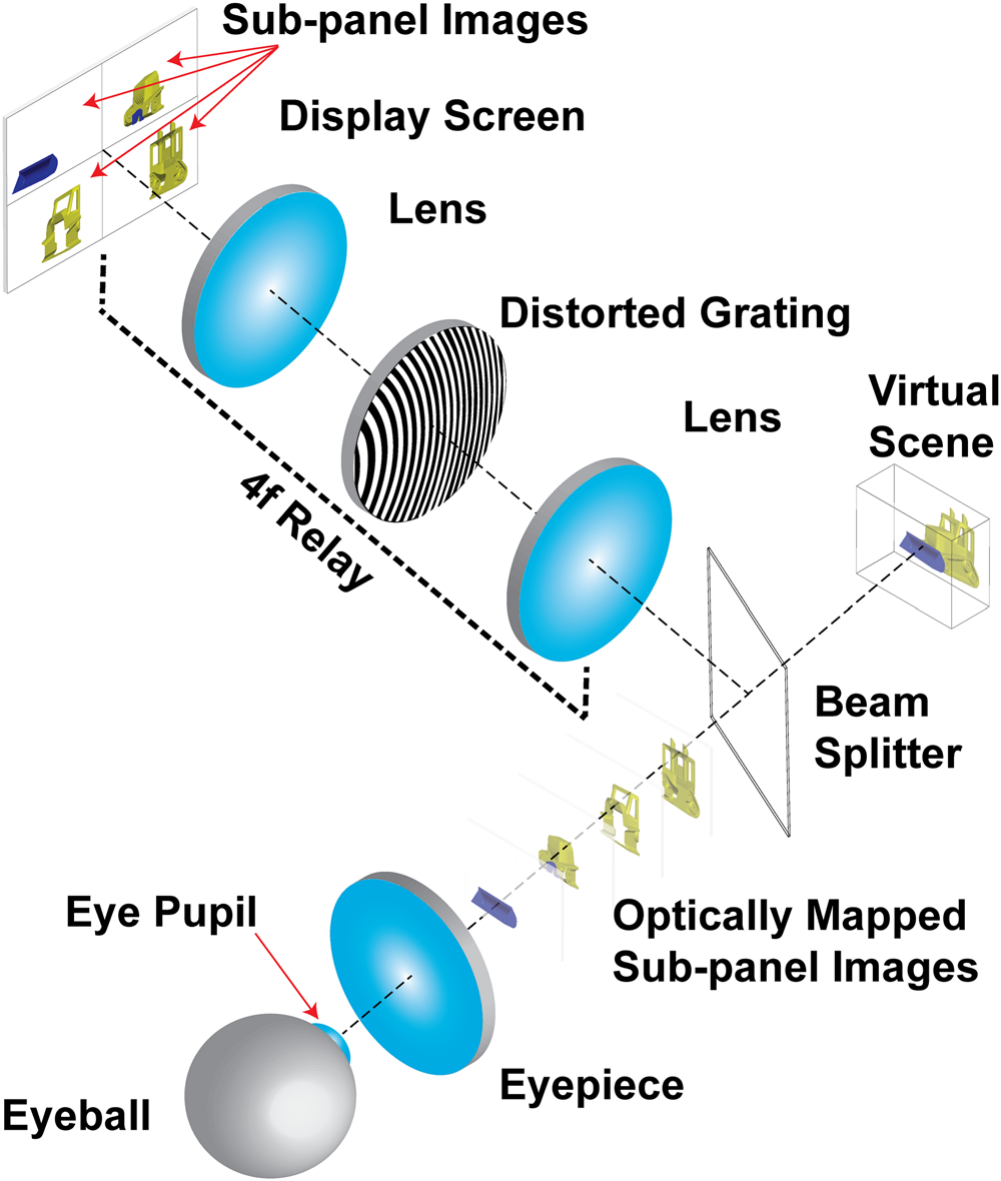
Operating principle.

We illustrate the operating principle of the ATOM display in Fig. 1. In the real-three-dimensional display mode, we divide the input screen into multiple sub-panels, each displaying a depth image. These images are then relayed by a 4 *f* system with a distorted grating at the Fourier plane. Acting as an off-axis Fresnel lens, the distorted grating adds both the linear and quadratic phase factors to the diffracted waves, directing the associated sub-panel images to a variety of depths while shifting their centers towards the optical axis. Seeing through the eyepiece, the viewer will perceive these sub-panel images appearing at different virtual depths. Also, by rendering the contents using a depth-blending algorithm^47^, we can provide continuous focus cues across a wide depth range.

Due to the division of the display screen, given *N* depth planes, the resolution of each depth image is therefore 1/*N* of the display screen’s native resolution, leading to a reduced field of view (FOV). To accommodate applications where a large FOV is mostly desired, we can transform the ATOM display into a high-resolution two-dimensional display simply by removing the distorted grating from the optical path and displaying a single plane image at the screen’s full resolution. This switching mechanism thus grants users a freedom to adapt the ATOM display for a specific task.

## Methods

**Figure 2:**
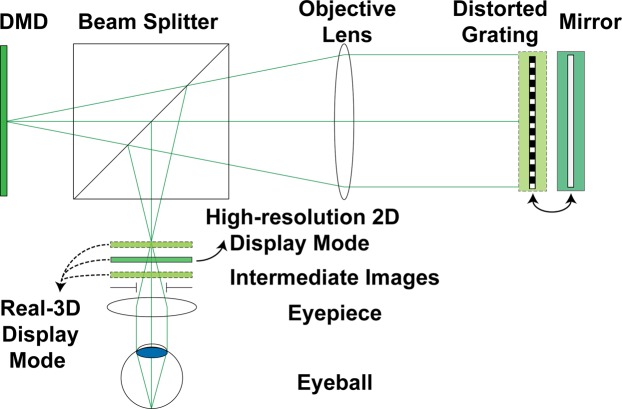
Optical schematic. DMD, digital mirror device.

We implemented the ATOM display in the reflection mode. The optical setup is shown in Fig. 2. At the input end, we used a green-laser-illuminated digital light projector (DLP4500, 912 × 1140 pixels, Texas Instruments) as the display screen. After passing through a beam splitter (50:50), the emitted light is collimated by an infinity-corrected objective lens (focal length, 100 mm; 2X M Plan APO, Edmund Optics). In the real-three-dimensional display mode, we place a reflective distorted grating at the Fourier plane—the back aperture of the objective lens—to modulate the phase of the incident light. While in the high-resolution two-dimensional display mode, we replace the distorted grating with a mirror. The reflected light passes through the objective lens and is reflected at the beam splitter, forming intermediate depth images (real-three-dimensional display mode) or a full-resolution two-dimensional image (high-resolution two-dimensional display mode) at the front focal plane of an eyepiece (focal length, 8 mm; EFL Mounted RKE Precision Eyepiece, Edmund Optics). The resultant system parameters for the high-resolution two-dimensional and real-three-dimensional display modes are shown in Table 1.

**Table 1:** System parameters of an ATOM display.

	Resolution (pixels)	FOV (degrees)
High-resolution two-dimensional display mode	900 × 900	63
Real-three-dimensional display mode	300 × 300	23

**Figure 3:**
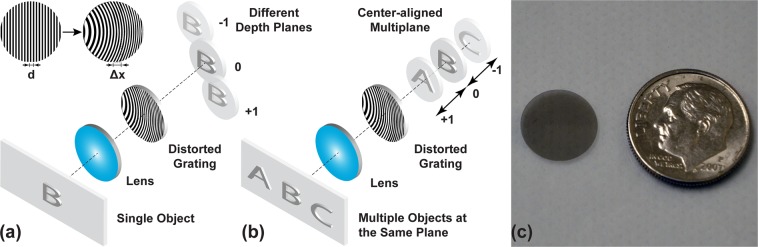
Image formation in a distorted-grating-based optical system. (**a**) Diffraction of a single object through a distorted grating. (**b**) Diffraction of multiple in-plane objects through a distorted grating. Only the on-axis diffracted images are illustrated. (**c**) Photograph of a distorted grating. A US dime is placed at the right for size reference.

As an enabling component, the distorted grating functions as a multiplexed off-axis Fresnel lens in the ATOM display. Although distorted gratings have been long used in microscopy^48^, wavefront sensing^49^, and optical data storage^50^, we deploy it for the first time in a display system. We elaborate the effect of a distorted grating on an optical system in Fig. 3(a). Given a single object, the distorted grating introduces varied levels of defocus to the wavefront associated with different diffraction orders. When combined with a lens, the distorted grating modifies its focal length and laterally shifts the image for non-zero diffraction orders. Similarly, given multiple objects located at the same plane but different lateral positions, the distorted grating can simultaneously project their different diffraction-order images onto various depths while maintaining their centers aligned (Fig. 3(b)).

The unique diffraction property above originates from the spatially-varied shift in the grating period, *Δ*_*x*_(*x*, *y*) (Fig. 3(a)). The correspondent local phase shift for diffraction order *m* can be written as:

1 $${\phi }_{m}(x,y)=\frac{2\pi m{{\Delta }}_{x}(x,y)}{d}+\frac{2\pi mx}{d},$$

where *d* is the period of an undistorted grating. At the right side of Eq. 1, the first and second term depict the contributions from the distorted and undistorted grating period, respectively. If the first distorted term has a quadratic form,

2 $${{\Delta }}_{x}(x,y)=\frac{{W}_{20}d}{\lambda {R}^{2}}({x}^{2}+{y}^{2}),$$

where *R* is the grating radius, and W is the defocus coefficient, and *λ* is the wavelength, the correspondent phase change ${\phi }_{m}^{Q}$ would be:

3 $${\phi }_{m}^{Q}(x,y)=m\frac{2\pi {W}_{20}}{\lambda {R}^{2}}({x}^{2}+{y}^{2}).$$

We can consider this phase change is contributed by a lens with an equivalent focal length,

4 $${f}_{m}=\frac{{R}^{2}}{2m{W}_{20}}.$$

The sign of diffraction order *m* thus determines the optical power of the distorted grating.

On the other hand, the second undistorted term in Eq. 1 introduces a linear phase shift to the wavefront in the form:

5 $${\phi }_{m}^{L}(x,y)=\frac{2\pi \,{\sin }\,\theta }{\lambda }x,$$

where *θ* is the diffraction angle, and it can be calculated from the grating equation:

6 $$d\,{\sin }\,\theta =m\lambda .$$

Under the small-angle approximation, we correlate the diffraction angle *θ* with the lateral shift ${l}_{{x}_{m}}$ of a sub-panel image in the ATOM display as:

7 $$\theta =\frac{{l}_{{x}_{m}}}{{f}_{OBJ}},$$

where *f*_*OBJ*_ is the focal length of the objective lens (Fig. 2).

Finally, combining Eqs 1–7 gives:

8 $${\phi }_{m}(x,y)=\frac{\pi ({x}^{2}+{y}^{2})}{\lambda {f}_{m}}+\frac{2\pi }{\lambda }\,{\sin }(\frac{{l}_{{x}_{m}}}{{f}_{OBJ}})x.$$

Notably, the phase pattern in Eq. 8 is inherently associated with diffracted depth images. By contrast, in the OMNI display^46^, to calculate the required phase pattern displayed at LCOS-SLM, we must perform optimization for each depth image, which is computationally extensive and may lead to an ill-posed problem when the number of depth planes increases.

In our prototype, we used only the +1, 0, and −1 diffraction orders and projected their associated images to 0, 2, 4 diopters, respectively. The correspondent focal lengths and diffraction efficiencies^51^ of this binary amplitude grating with 50% duty cycle were computed and shown in Table 2. We calculated the structural parameters of the distorted grating (Table 3) and fabricated it as a reflective mask using direct laser writing on a soda lime base with high reflective chrome coating (Fig. 3(c)).

**Table 2:** System parameters of an ATOM display.

Diffraction order	+1	0	−1
Dioptric depth (diopter)	0	2	4
*f*_*m*_ (m)	81.4	Inf.	−81.4
Diffraction efficiency (%)	10.1	25	10.1

**Table 3:** Structural parameters of the distorted grating.

*W*_20_ (nm)	*d* (μm)	*R* (mm)
185.8	43.2	5.5

### System Evaluation

**Figure 4:**
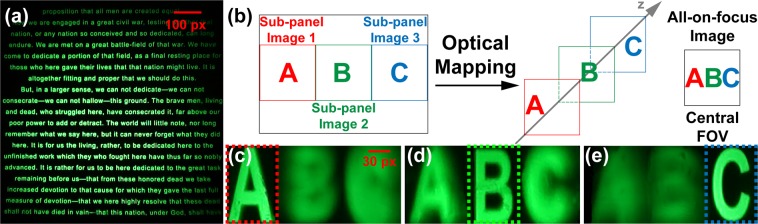
Evaluation of display performance. (**a**) Image captured in the high-resolution two-dimensional display mode. (**b**) Optical mapping in the real-three-dimensional display mode. (**c**–**e**) Images captured at three depths. px, pixels.

To demonstrate the high-resolution two-dimensional display, we captured a representative image at the intermediate image plane using a Sony Alpha 7S II digital camera (Fig. 4(a)). To evaluate the real-three-dimensional display performance, we carried out a simple depth mapping experiment. We displayed three letters “A”, “B”, “C” on the three sub-panels of the display screen respectively (Fig. 4(b)) and captured the remapped images at three designated depth planes (0, 2, and 4 diopters). To compensate for the intensity variation between 0 and ±1 diffraction-order images, we applied a neutral density filter to the central sub-panel image to dim its brightness. The remapped letter images are shown at three designated depths (Fig. 4(c–e)), respectively. The letters are in focus at their designated depths while blurred at other depth locations.

**Figure 5:**
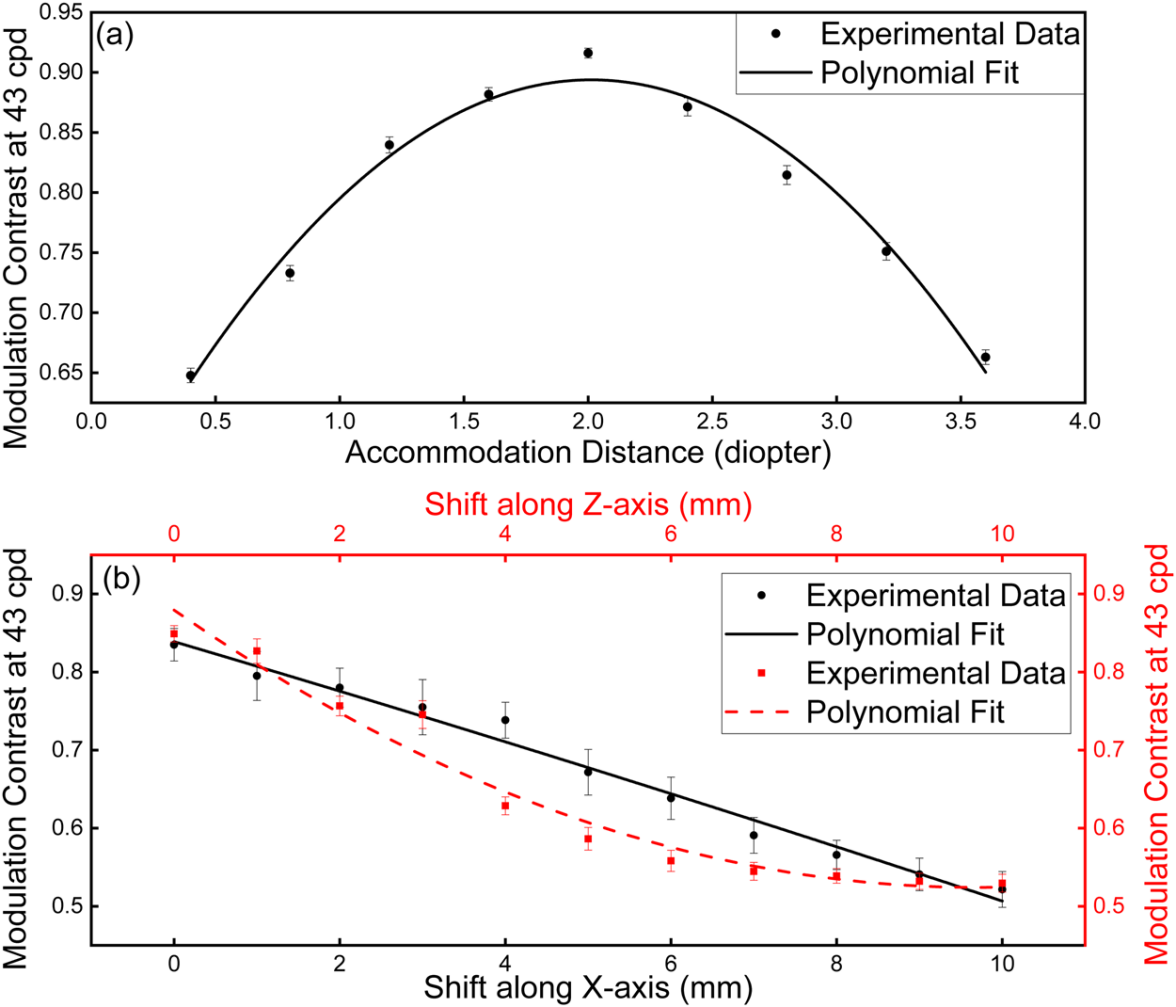
System evaluation. (**a**) Assessment of focus cues. (**b**) Sensitivity of modulation contrast to the relative shift of the distorted grating.

To assess the focus cues provided by the ATOM display, we adopted a standard two-plane verification procedure^47^ and measured the modulation transfer function (MTF) at different accommodation distances. We directly placed the camera at the nominal working distance of the eyepiece and varied its axial position to mimic the accommodation of an eye. Two identical sub-panel images (slanted edge) were displayed at the input screen and projected to depth planes at 0 and 4 diopters with their centers aligned. The target depth was rendered at the dioptric midpoint 2 diopters position by using a linear depth-weighted blending where the image intensity at each designated depth plane is proportional to the dioptric distance of the point from that plane to the viewer along a line of sight. The experimentally-measured accommodation is 2.05 diopters, closely matching with the target value (Fig. 5(a)).

Next, we tested the system’s stability during the mechanical switch between two display modes. To characterize the tolerance of the distorted grating to the misalignment, we varied the distorted grating’s position both laterally and axially and measured the correspondent display performance. Again, we chose MTF as the metric and used the dual-plane characterization method above. The results imply that the MTF decreases as the grating’s position shift increases (Fig. 5(b)). Here the position shift is calculated with respect to the grating’s nominal position. Given a threshold Δ*MTF* = 0.1, the system can tolerate a shift of 2 mm along both lateral and axial axes. This relatively loose tolerance favors the low-cost production of the device.

Finally, we demonstrated the system’s capability in displaying a complex three-dimensional scene. Herein a linear depth-weighted blending algorithm was applied to generate different sub-panel images^52^. In a nutshell, the image intensity at each depth plane is linearly proportional to the dioptric distance between that plane and the viewer. Meanwhile, the sum of the image intensities is kept a constant at all depth planes. To achieve uniform image brightness across the entire depth range, we applied a tent filter to the linear depth blending, where the light intensity for each depth plane reaches a maximum at its nominal position and minimum elsewhere.

**Figure 6:**
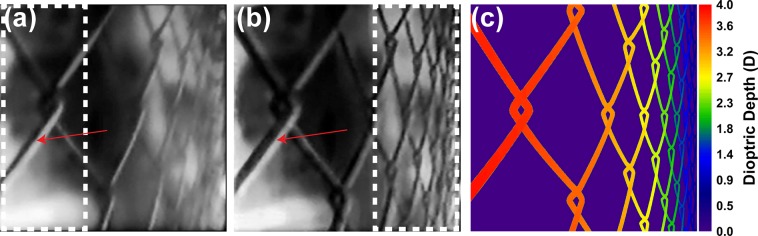
ATOM display of a complex three-dimensional scene. Representative depth images captured at (**a**) near-end (4 diopters) and (**b**) far-end (0 diopter). (**c**) Ground-truth depth map. The line spreads at the arrow-pointed locations in (**a**) and (**b**) are 70 and 120 pixels, respectively.

Based on the algorithm above, we generated the sub-panel images for three designated depth planes (0, 2, and 4 diopters) for a three-dimensional image (a tilted fence) and displayed them at the input screen. A camera was placed in front of the eyepiece with its focal depth adjusted to mimic the eye’s accommodation. At a series of depths, the images were captured accordingly. The representative depth-fused images at near-end (4 diopters) and far-end (0 diopter) are shown in Fig. 6(a,b), respectively, closely matching the ground-truth depth map (Fig. 6(c)). To quantitively assess the focusing effect, we measured the line spread of the fence image at the arrow-pointed location. The corresponding values at the near-focus and far-focus are 70 pixels and 120 pixels, respectively.

## Discussion and Conclusion

In summary, based on the human-centric design principle, we developed a compact ATOM near-eye display which can provide correct focus cues that best mimic the natural response of human eyes. By projecting images of different sub-panels at a two-dimensional display screen to designated depths using a distorted grating, we created a real three-dimensional image covering the depth range from 4 diopters to infinity. The employment of all-passive optical components reduces the system dimension and power consumption, thereby improving the system’s wearability.

Due to the division of the input screen panel, each depth image has a reduced pixel resolution, a common problem for the spatial-multiplexing-based approaches^46,53^. To alleviate this problem, we built the system on a transformable architecture—we can switch between a high-resolution two-dimensional display mode and a multiple-plane three-dimensional display mode simply by removing or adding the distorted grating into the optical path, thereby providing task-tailored viewing experiences. Moreover, we envision this problem can be further reduced by using an ultra-high-resolution (4k or 8k) micro-display panel at the input end—after division, each sub-panel image still possesses a high pixel resolution. Fortunately, such ultra-high-resolution micro-display panels have gradually become available to the market.

Although not demonstrated, we can enable more depth planes by using a distorted grating with periodic structures along two dimensions^54^. Using such a two-dimensional diffractive element, we can perform lateral optical mapping along both *x* and *y* axes, leading to a more efficient utilization of screen pixels. In the ideal case, the total number of remapped pixels is equal to that of the original display screen. For example, given an input screen of *N* × *N* pixels, an ATOM display with a two-dimensional distorted grating can project a total of nine depth images, each with *N/*3 × *N*/3 pixels and associated with a unique diffraction order (*L*_*x*_, *L*_*y*_), where *L*_*x*_, *L*_*y*_ = 0, ±1.

In the current ATOM display prototype, we decrease the light intensity associated with 0 diffraction order to compensate for the difference in diffraction efficiency, however, at the expense of reduced light throughput. To fully utilize the dynamic range of the display screen, rather than using a binary-amplitude distorted grating, we can employ a sinusoidal-phase distorted grating^51^ and build the system in the transmission mode. Such a diffractive phase element allows an approximately uniform energy distribution among ±1 and 0 orders, and it can be holographically fabricated by creating an interference pattern on a photoresist.

## Data Availability

The data that support the findings of this research project are available from the corresponding author upon request.
